# Concurrent validity of a new screening test to evaluate the information processing speed

**DOI:** 10.1590/1980-5764-DN-2024-0183

**Published:** 2025-01-27

**Authors:** Camila Fernanda Cabrera Zavala, Oscar Agustín Porta, Alén Martín, Edison Vega Valderrama, María del Rosario Lis, Wilson Gualotuña Pachacama, Diana Soto

**Affiliations:** 1 National University of Comahue, Faculty of Medical Sciences, Carlos G. Durand General Acute Hospital, Buenos Aires Autonomous City of Buenos Aires, Argentina.; 2 University of Buenos Aires, Faculty of Medicine, Carlos G. Durand General Acute Hospital, Buenos Aires Autonomous City of Buenos Aires, Argentina.; 3 National University of Comahue, Faculty of Agrarian Sciences, Carlos G. Durand General Acute Hospital, Buenos Aires Autonomous City of Buenos Aires, Argentina.; 4 Carlos G. Durand General Acute Hospital, Buenos Aires Autonomous City of Buenos Aires, Argentina.

**Keywords:** Neuropsychological Tests, Wechsler Scales, Locomotion, Educational Status, Testes Neuropsicológicos, Escalas de Wechsler, Locomoção, Escolaridade

## Abstract

Information processing speed (IPS) evaluates the time at which a person reacts to a stimulus. The Wechsler for Adults-III (WAIS-III) includes this domain in the digit symbol test (DS-T).

**Objective:**

The aim of this study was to validate a new screening test that could measure the IPS in association with the individual’s age and scholarship.

**Methods:**

A new instrument to measure the IPS was constructed, automatic cashier test (AC-T), and for its validation, the DS-T was also carried out.

**Results:**

The media of time use in the ACT was 12.3 s; the media of hits in the DS-T was 38.8, with a p<0.0001 and R^2^: 0.40.

**Conclusion:**

P-value showed a linear association between the two tests, but the R^2^ results showed a low association between these. In the same way, the correlation between both tests is promising, since it shows that both are tests that measure the IPS in a different way with different constructs such as the verbal fluency test that is also used to evaluate IPS. On the other hand, both tests exposed that the scholarship positively influences the IPS.

## RESUMO

A velocidade de processamento da informação (IPS) avalia o tempo em que uma pessoa reage a um estímulo. O Wechsler for Adults-III (WAIS-III) inclui esse domínio no Digit Symbol Test (DS-T).

**Objetivo:**

Validar um novo teste de triagem capaz de medir o IPS em associação com a idade e a escolaridade do indivíduo.

**Métodos:**

Um novo instrumento de medida do IPS foi construído: o Automatic Cashier Test (ACT), e para sua validação também foi realizado o DS-T.

**Resultados:**

A média de uso do tempo no ACT foi de 12,3 segundos, e a média de acertos no DS-T, de 38,8, com P<0,0001 e R2=0,40.

**Conclusão:**

O valor-p mostrou uma associação linear entre os dois testes, mas os resultados do R^2^ revelaram baixa associação entre eles. Da qualquer forma, a correlação entre os dois testes é promissora, pois mostra que ambos são testes que medem o IPS de maneira diferente com construtos diferentes, como o teste de fluência verbal, que também é usado para avaliar o IPS. Em contrapartida, os dois testes utilizados expuseram que o *scholarship* influencia positivamente o IPS.

## INTRODUCTION

Information processing speed (IPS) is a general ability of the cognitive system, known as the time in which the organism receives a stimulus, processes it, and prepares a response to it^1^. It has been shown that there is a relationship between IPS and the cognitive performance of individuals^2^, being considered one of the general mental capacities of the cognitive system, which can be modified with the individual’s age^3^ and in association with particular previous life experiences^4^.

Once the stimulus has been received and translated by the sensory organs, the speed at which a problem is solved requires the ability of a cognitive organization to carry out a search for strategies to focus attention and activate selection processes that finally allow it to find the motor programs that will be converted into actions through the locomotor system to solve the problem of interest^5^.

Currently, there is a large number of cognitive tests that include this variable in their evaluation, such as the Stroop test^6^, the symbol search, and the number key subtests of the Wechsler Intelligence Scale for Adults-III (WAIS-III), the last one of these, with its adaptation in Argentina^7^.

However, these tests require great concentration and collaboration from the subject being evaluated, which can often be altered by external factors such as a noisy or uncomfortable environment, physical or mental fatigue, taking into account that especially adult patients often have associated systemic pathologies, external to cognitive factors such as osteoarthritis or arthralgias that limit the time at which they can remain in the same position or perform accurate movements such as drawing, due to the imposition of a locomotor and not a cognitive difficulty.

In the WAIS digit symbol test (DS-T), the individual must translate a numerical key into symbols and draw on a grid each of these new symbols under their corresponding number. The patient is given 120 s to complete it and then is quantified to identify how many digits it was able to translate correctly. In this way, the number of correct answers in this period of time is evaluated, and the final value is taken as a representative result of the IPS^6^.

In relation to these, we present the design of a new test to evaluate the IPS, also using a unit of time that allows us to evaluate the speed component of the IPS, but minimizing the influence of the mechanical conditioning of the locomotor system on the final value of the test, since in this new test we propose to perform a very simple locomotor movement as opposed to the creation of a complex figure proposal in the DS-T, prioritizing the value of the cognitive processes and attention in the solving of the problem.

In addition to this, it should also be considered that conventional tests do not include, within the determinants of their results, the subjects under study’s level of scholarship, being certain that the cognitive domains can be modified by experience and the external environment in which the individuals develop their life^8^.

The intention of validating this new test is to associate it with another battery of tests already validated in Argentina such as the Mini-Mental^9^ and the MOCA^10^ test, thus having screening tests that are easy to execute and quick to apply in the consultation, being able to use them in a standardized way in annual controls in patients who come for a neurological follow-up for some other underlying pathology that can also affect cognitive domains in their progression, such as multiple sclerosis, epilepsy, and cerebrovascular diseases.

Having stated this, this work sets as its objective the validation of a new screening test that evaluates the speed of information processing in an efficient way, with fewer resources, that can adjust to the level of schooling of the individual at issue, and that has standardized values for the Spanish-speaking population residing in the metropolitan area of the City of Buenos Aires.

## METHODS

**Figure 1:**
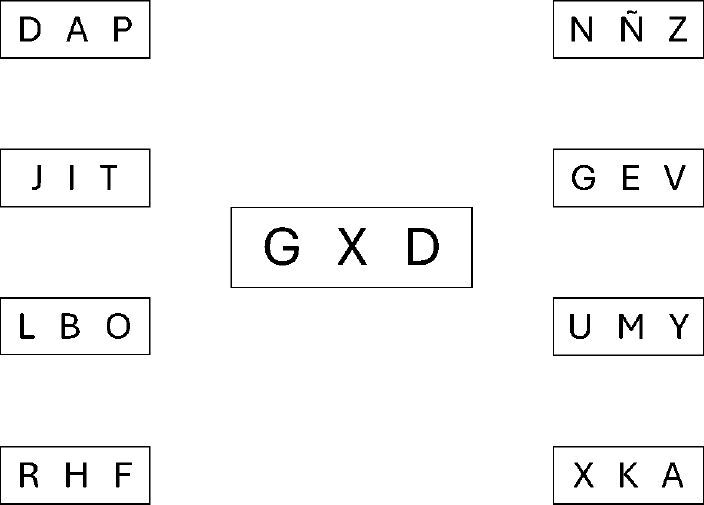
Automatic cashier test (AC-T).

An instrument to measure IPS was developed based on a preliminary trial^8^, which consisted of four printed sheets (one of them as a simulacrum), each sheet had an alphabetical code of three letters arranged in the center of it, surrounded on both sides by four rectangles, with another alphabetic code also of three different letters from each other and with respect to the code of the central line, looking for similarity with the alphabetical verification key of the AC-T. The subject had to recognize the central letters on each sheet and place them on the side columns, selecting them with a pen. It was decided to replicate the test three times in order to reduce the error in the measurement. For each slide, the time was taken in seconds, and then the media of the total time to complete the three sheets was obtained. This test was called automatic cashier test (AC-T) (Figure 1).

In this new instance, in order to validate this method, it was decided to compare it with the WAIS-III DS-T, adapted in Argentina, asking patients to perform the two tests.

There were excluded subjects aged under 55 or over 84, those who had a previous diagnosis with neurodegenerative diseases such as Parkinson’s or Alzheimer’s disease, demyelinating diseases such as multiple sclerosis, patients with a history of cerebrovascular event, those who had severe sensory disorders, and those who were institutionalized or on psychopharmacological treatment with antidepressants or antipsychotics.

The age variable was segmented by selecting age groups according to the WAIS scales with the final result of five categories presented: 55–64, 65–69, 70–74, 75–79, and 80–84 years.

There were evaluated patients aged between 55 and 84 years old, who attended the Durand Hospital for neurology consultation and other specialties such as geriatrics, cardiology, medical, clinical, hematology, and traumatology.

All participants spoke Spanish and lived in the metropolitan area of Buenos Aires, and the age and schooling variables were analyzed.

A total of 139 patients were studied, and then we corrected the dispersions of this number using an influencing point analysis, eventually including finally 130 patients.

The association between the AC-T and the Wais-III DS-T was evaluated using a linear regression model.

## RESULTS

Data were collected from a total of 139 patients, divided into five selected age groups, to whom a statistical adjustment called influential point analysis was applied to eliminate those extreme values, which due to their atypical presentation could have altered the slopes of the curves, including in the final work a data of 130 patients.

The value of the media total time to solve the AC-T was 12.3 s (±5.5), while the value of the media of correct answer in the DS-T was 38.8 (±15.7), with a p<0.0001 and R^2^ of 0.40.

P-value was significant in Groups 1 (P=0.0001), 2 (P=0.0025), 3 (P=0.0003), and 5 (P=0.0036), with a non-significant p-value, but showing a tendency toward linear association in Group 4 (P=0.0685).

**Figure 2:**
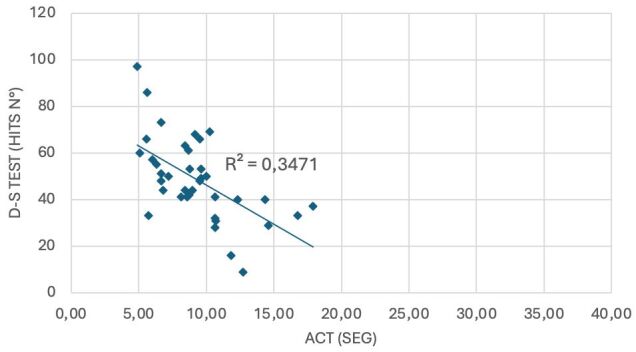
DS-T hits based on seconds spent in AC-T for Group 1 (55–64 years).

**Figure 3:**
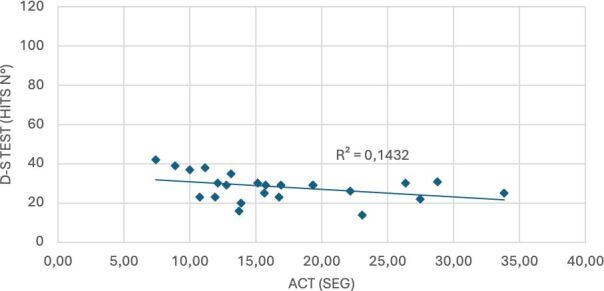
DS-T hits based on seconds spent in AC-T for Group 4 (75–79 years).

The R^2^ values were: Group 1: 0.34, Group 2: 0.58, Group 3: 0.39, Group 4: 0.14, and Group 5: 0.58 (Figures 2 and 3).

The studied group had a level of schooling consisting mostly of primary or secondary education and, to a lesser extent, tertiary and university studies.

The specific data obtained for each age group are presented in Tables 1 and 2.

**Table 1:** Number of patients, time in ACT, hits in DS-T by age, p-value, and regression coefficients for linear regression model.

Age group	n	Time	Hits	p-value	R^2^
1 (55–64 years)	38	9.3±3.1	48.6±17.6	<0.0001	0.34
2 (65–69 years)	28	11.9±4.6	37.2±14.1	0.0025	0.58
3 (70–74 years)	28	11.6±4.9	36.7±14.0	0.0003	0.39
4 (75–79 years)	24	16.9±6.8	28.1±6.9	0.0685	0.14
5 (80–84 years)	12	14.5±5.4	29.5±11.0	0.0036	0.58
Total	130	12.3±5.5	38.0±15.7	<0.0001	0.40

**Table 2:** Percentage of scholarship by age group and distribution by sex.

Group Nº	Female (%)	Male (%)	Primary (%)	Secondary (%)	Tertiary (%)	University (%)	Other
1 (55–64 years)	62.16	34.84	28.20	35.90	12.80	23.10	x
2 (65–69 years)	63.33	36.66	22.60	38.00	12.90	25.80	x
3 (70–74 years)	79.31	20.70	31.00	48.30	10.30	10.30	x
4 (75–79 years)	38.91	37.04	25.90	51.00	14.80	3.70	3.70% non-scholarship
5 (80–84 years)	69.23	30.77	23.00	43.10	7.70	23.00	x

With respect to the prevalence of the clinical condition of these patients, taking into account the exclusion criteria, for all the groups the most prevalent disease was arterial hypertension.

## DISCUSSION

The results of the R^2^ currently show an insufficient association between the AC-T and the DS-Test.

If we analyze the two tests, we can highlight that, even though both tests take into account the variable “time” in their measurement, in the DS-T, the time is a fixed variable. Whereas, in the new test, the AC-T, time is modified according to the operator’s abilities to solve the test and finally constitutes a determining value of the test, which is its final result, taken as an objective and representative value of the test. This generates difficulties when adjusting the fixed time variable of the 120 s from the DS-T, with the seconds used to solve the ACT, whose media resolution was about 12.3 s, a small proportion of the total 120 s of the test from which it is compared and sought to validate.

In the ACT, the variable “time” is not a fixed variable, and its seconds vary widely depending on the particular skills of the different subjects who solve the test. To bridge this gap, it could be postulated that more sheets should be added to the new test, thus increasing the time taken to solve it and bringing it closer to the measurement of the second employees in the WAIS, with the disadvantage of making the test in question more complex and moving it away from its main objective of being a screening test.

Furthermore, it is also pertinent to consider that both tests could be aimed at evaluating different constructs. On one hand, the AC-T test does not impose the challenge recourse of free recall, where the ability to quickly learn new symbols imposes an advantage at the moment of solving the test, while it uses alphabetic symbols that are already known to the subjects evaluated, obviating the implication of including the evocation of free memory in the evaluation.

In this regard, Teresa Simón et al.^11^ and Naveh-Benjamin et al.^12^ studied the impact of the loss of the ability of free recall in older patients, as also evidenced in our study. We observed a decrease in the hit rates of the DS-T as the age groups increased. This decline is consistent with the deficit in working memory often seen in older individuals. Such deficits can pose challenges when recalling the PDS series of symbols.

This new test also leaves aside the impact of graphomotor skills since it only imprints the need to select, with a pen, the alphabetic symbol presented and does not evaluate the ability to replicate figures as the DS-T does, resorting to the complexity of activation of neural networks that this skill implies.

Research has shown that fine motor skills influence gross motor skills and affect the speed at which children perform various tasks as they develop cognitively^13^. In this regard, Spanish authors conducted a study with 2,564 patients. They utilized different combinations of cognitive tests and visuoperceptual exercises, highlighting the relationship between visuomotor coordination and executive functions in determining how quickly tasks are completed^14^.

On the other hand, in the general analysis of the data presented in Tables 1 and 2, we observed that in all five groups, the percentage of patients with tertiary and university education was low. The secondary level education represented the highest percentage of formal educational status reached in the five groups. This fact was also influenced by the sociodemographic characteristics of the studied population, considering that these patients were recruited from public healthcare services.

Now, starting from the fact that the group with the lowest percentage of patients with university education was Group 4, we could find that this group also had the lowest performance in the final results of both tests. This demonstrates that those groups with a higher percentage of members with university or tertiary education achieved better results in the measuring of the tests’ IPS, indicating that educational level positively influences this domain, regardless of the test used.

In conclusion, it is pertinent to highlight that, although the slope of the regression curves between the results of the AC-T and DS-T variables was significant, the level of association estimated from the R^2^ indicator ranged from 0.34 to 0.58, below the recommended level (between 0.8 and 0.9).

From this initial study, the AC-T should be adjusted, and eventually, new constructs should be added to make this new test compatible with the DS-T. The data collected from WAIS-III will be used to expand its data in future perspectives as well as to build a local Argentine scale adjusted to scholarship.

In addition to the scholarship, both tests demonstrated that patients who reached a higher educational level obtained better results in the two tests. This not only indicates that the educational level positively influences the IPS but also suggests that both tests measure a similar underlying construct.

The collected data could be further utilized to not only adjust the WAIS according to the educational level but also to tailor the AC-T, aiming to design a test that accurately measures IPS in relation to scholarships. Currently, there is a lack of validated tests in our country.

Although it is true that the association between the two tests yielded a different value than expected, the weak correlation between both tests is promising. It indicates that both tests assess IPS in different ways and through distinct constructs, such as the verbal fluency test, which is also used to evaluate IPS.
